# ERAP1 is a critical regulator of inflammasome-mediated proinflammatory and ER stress responses

**DOI:** 10.1186/s12865-022-00481-9

**Published:** 2022-03-04

**Authors:** Maja K. Blake, Patrick O’Connell, Yuliya Pepelyayeva, Sarah Godbehere, Yasser A. Aldhamen, Andrea Amalfitano

**Affiliations:** 1 Department of Microbiology and Molecular Genetics, College of Osteopathic Medicine, Michigan State University, East Lansing, MI USA; 2 Department of Pediatrics, College of Osteopathic Medicine, Michigan State University, East Lansing, MI USA

**Keywords:** Innate immunity, Inflammasome, Ankylosing spondylitis, ER stress

## Abstract

**Background:**

In addition to its role in antigen presentation, recent reports establish a new role for endoplasmic reticulum aminopeptidase 1 (ERAP1) in innate immunity; however, the mechanisms underlying these functions are not fully defined. We previously confirmed that loss of ERAP1 functions resulted in exaggerated innate immune responses in a murine in vivo model. Here, we investigated the role of ERAP1 in suppressing inflammasome pathways and their dependence on ER stress responses.

**Results:**

Using bone marrow-derived macrophages (BMDMs), we found that loss of ERAP1 in macrophages resulted in exaggerated production of IL-1β and IL-18 and augmented caspase-1 activity, relative to wild type macrophages. Moreover, an in vivo colitis model utilizing dextran sodium sulfate (DSS) confirmed increased levels of proinflammatory cytokines and chemokines in the colon of DSS treated ERAP1^−/−^ mice as compared to identically stimulated WT mice. Interestingly, stimulated ERAP1^−/−^ BMDMs and CD4^+^ T cells simultaneously demonstrated exaggerated ER stress, assessed by increased expression of ER stress-associated genes, a state that could be reverted to WT levels with use of the ER stress inhibitor Tauroursodeoxycholic acid (TUDCA).

**Conclusions:**

Together, these results not only suggest that ERAP1 is important for regulating inflammasome dependent innate immune response pathways in vivo, but also propose a mechanism that underlies these changes, that may be associated with increased ER stress due to lack of normal ERAP1 functions.

**Supplementary Information:**

The online version contains supplementary material available at 10.1186/s12865-022-00481-9.

## Introduction

Polymorphisms in endoplasmic reticulum aminopeptidase 1 (ERAP1) have been linked to several important autoimmune diseases such as Ankylosing Spondylitis (AS), Type I diabetes, Multiple Sclerosis, and Behçet's disease, suggesting that causation of these conditions may share a common mechanism due to dysfunctions in ERAP1 [1]. ERAP1 not only plays a canonical role in the adaptive immune system via its role in the ER as an aminopeptidase processing peptides destined for MHC I presentation to CD8^+^ T cells [2–4], but ERAP1 functions are also required to suppress a number of proinflammatory, innate immune responses [5–8].

We previously demonstrated an important regulatory role for ERAP1 in innate immunity by showing that ERAP1^−/−^ mice elicit exaggerated innate immune responses following exposure to several pathogen-derived components [5, 6]. TLR-stimulated ERAP1^−/−^ mice produced significantly higher levels of multiple Th-1 skewing proinflammatory cytokines and chemokines such as MCP-1, RANTES, IL-12p40, and IL-12p70, as compared to WT mice [5]. These exaggerated innate immune responses positively correlated with enhanced activation of innate (natural killer (NK) cells and dendritic cells (DCs)) as well adaptive immune cells in ERAP1^−/−^ mice [5, 7]. Several other independent studies have also indicated a critical role for ERAP1 in innate immune cell regulation [9–11]. We have also demonstrated that human ERAP1 variants caused enhanced IL-1β production from human immune cells [6] in a mechanism that involves K+ efflux [12], which is a well-known signal of NLRP3 inflammasome activation. However, the underlying mechanisms responsible for these ERAP1-dependent immune responses remain unknown.

Synthesis of inflammatory cytokines and chemokines by the innate immune system is primarily initiated by activation of various germline-encoded pattern-recognition receptors (PRRs), such as Toll-like receptors (TLRs), RIG-I-like receptors (RLR), and NOD-like receptors (NLRs) [13]. Activation of these PRRs drive the coordinated activation of intracellular signaling pathways that regulate the transcription of inflammatory cytokine and chemokine genes, as well as other innate immune defense responses [13]. For example, activation of caspase-1 by NLRP3, and other caspase-1-activating inflammasomes, results in the processing and release of the proinflammatory cytokines IL-1β and IL-18 [14, 15]. Upon binding to their receptors and following recruitment of the MYD88 adaptor, IL-1β and IL-18 induce the expression and production of several proinflammatory cytokines and chemokines [14, 15]. Interestingly, AS patients are known to have increased proinflammatory cytokine levels, including IL-1β [16].

Notably, inflammatory bowel disease has also been directly and indirectly associated with ERAP1 linked diseases, for example gut inflammation was found in 25–49% of AS patients [16]. We previously demonstrated that ERAP1^−/−^ mice not only have exaggerated innate and adaptive immune responses to various stimuli, but also demonstrate severe intestinal inflammation upon DSS treatment, with a phenotype paralleling patients with AS [8]; however, the mechanisms underlying responsible for ERAP1’s role in modulating these inflammatory responses have also not been identified.

Here, we hypothesized that ERAP1 may have an intrinsic immune regulatory function in innate immune cells in addition to its normal role in antigen trimming in the ER. We investigated the impact that ERAP1 functions have on inflammasome and TLR receptor regulation ex vivo using a BMDM-based model for analyzing inflammasome and ER stress responses, and in vivo by utilizing the DSS-induced intestinal inflammation model. Our results suggest that deletion of ERAP1 aminopeptidase activity not only impacts cellular display of MHC-I peptides, but is correlated with the activation of multiple innate immune pathways and predisposes cells to increased ER stress, all phenomenon that are also present in several ERAP1-linked autoimmune diseases [17–19].

## Materials and methods

### Ex vivo cell culture

Bone marrow cells were extracted from the femurs of male 6–8 weeks old WT and ERAP1^−/−^ mice, and red cells were removed using ACK lysis buffer (Invitrogen, Carlsbad, CA), as previously described [20]. Bone marrow cells were cultured in Dulbecco’s Modified Eagle Medium (DMEM) supplemented with 10% fetal bovine serum (FBS), 1% penicillin–streptomycin–fungizone, and 30% supernatant derived from confluent L929 cell cultures. At day 7, immature macrophages (5 × 10^5^ cells) were collected and plated in a 24-well plate and primed with LPS (20 ng/mL) for 12 h or primed and pre-treated with TUDCA (500 µg/mL) for 12 h. Cells were stimulated for another 16 h with heat-killed listeria monocytogenes (HKLM) (10^8^ cells/well), lipopolysaccharide (LPS) (1 μg/mL), monophosphoryl lipid A (MPLA) (1 μg/mL), CpG oligodeoxynucleotides CpG ODN (2.5 ug/mL), polyriboinosinic: polyribocytidylic acid (poly(I:C)) (10 μg/mL), nigericin (10 μg/mL), muramyl dipeptide (MDP) (10 μg/mL), flagellin (250 ng/mL), or alum (1 mg/mL) (all from InvivoGen, San Diego, CA, USA). Stimuli concentrations were based on manufacturer’s guidelines. For AIM-2 inflammasome activation, cells were transfected with poly(dA:dT) (8 μM) (InvivoGen, San Diego, CA, USA) using Lipofectamine 2000 (Life Technologies Inc.) according to manufacturer's protocol. For MSU crystal stimulation, BMDMs were primed with LPS (15 ng/mL) and stimulated with 100 ug/mL MSU crystal (InvivoGen, San Diego, CA) for approximately 18 h. Cell supernatants were collected and used for ELISA and BioPlex assays.

### Cell staining and flow cytometry

Bone marrow cells were harvested and differentiated into macrophages, followed by stimulation with various NLRs and TLRs agonists. BMDMs were stained with either APC-conjugated anti-CD86, Pacific Blue CD80, APC MHC-I, PE MHC-I, PE-Cy7 CD11b, or V450 CD4. Cells were incubated with antibodies for 45 min on ice and washed with FACS buffer. For caspase-1 activity, FAM-FLICA in vitro caspase-1 detection kit (Immunochemistry Technologies, Bloomington, Minn., USA) was used and caspase-1 activity was measured, according to the manufacturer’s protocol. For p-p65 activation, BD Phosflow Perm Buffer (BD Biosciences, San Jose, CA) was used with Alexa-Fluor 647 p-p65 (Cell Signaling, Danvers,MA) staining, and measured with flow cytometry. A BD LSR II and Cytek Aurora instrument were used for data collection and data were analyzed using FlowJo software.

### Cytokine measurements following DSS treatment

To induce intestinal inflammation, mice were treated with 3% DSS (molecular mass 36–40 kDa; MP Biologicals) dissolved in sterile distilled water. Mice were given DSS in sterile drinking water ad libitum for experimental days 1–7 (controls received sterile water only) followed by regular water until the end of the study. DSS solutions were made fresh on day 0 and 3 and left until day 7. After 7 days of DSS treatment, colons were excised and a one centimeter piece of distal colon was homogenized mechanically in PBS containing 1% NP-40 and complete protease inhibitor cocktail (Roche). Protein concentrations were determined by BCA assay. Protein (100 μg) isolated from distal colons of naïve and DSS-treated mice was used in a 23-plex multiplex cytokine assay. The assay was performed according to the manufacturer’s instructions (Bio-Rad, Hercules, CA) via Luminex 100 technology (Luminex, Austin, TX), as previously described [5]. In vitro cell supernatant cytokines were measured with the same 23-plex multiplex cytokine assay following LPS priming (20 ng/mL), LPS treatment (100 ng/mL), Nigericin (10 ug/mL), and TUDCA (500 ug/mL). Stimuli concentrations were based on manufacturer’s guidelines.

### Animals

Mice deficient in ERAP1 and C57BL/6 WT mice were maintained at Michigan State University. Groups of mice were age- (8 to 10 weeks) and sex-matched. All animal procedures were reviewed and approved by the Michigan State University EHS, IBC, and IACUC and conformed to NIH guidelines (AUF number: 02/13-045-00).

### Quantitative RT-PCR

To determine relative levels of ER stress related genes, 2 × 10^6^ BMDMs were incubated with 25 ng/mL of LPS for 18 h and RNA was isolated using TRIzol reagent (Invitrogen, Carlsbad, CA, USA) according to the manufacturer’s protocol. BMDMs were pre-treated with an ER stress inhibitor, TUDCA (500 µg/mL), for 48 h prior to RNA extraction. To determine ER stress genes in CD4^+^ T cells, splenocytes were isolated from 5 WT and 4 ERAP1^−/−^ mice and CD4^+^ cells were isolated using negative selection (EasySep Mouse CD4^+^ T cell Isolation Kit). Cells were plated at 2 × 10^6^ and stimulated with 20 ng/mL LPS for 12 h. RNA was isolated by TRIzol reagent. Following RNA isolation, reverse transcription (RT) was performed on 180 ng of total RNA using SuperScript II (Invitrogen) RT and random hexamers (Applied Biosystems, Foster City, CA, USA) per manufacturer’s protocol. Primers were designed using Primer Bank web-based software (http://pga.mgh.harvard.edu/primerbank/). The primers that were used are as follows: ATF6: Forward: 5′TGGAGCAGGATGTCCCGTT3′, Reverse: 5′CTGTGGAAAGATGTGAGGACTC3′; ATF4: Forward: 5′CCTGAACAGCGAAGTGTTGG3′; Reverse: 5′TGGAGAACCCATGAGGTTTCAA3′; BiP: Forward: 5′CGAGGAGGAGGACAAGAAGG3′; Reverse: 5′CACCTTGAACGGCAAGAACT3′; XBP1spl: Forward: 5′TGCTGAGTCCGCAGCAGGTG3′ and Reverse: 5′GCTGGCAGGCTCTGGGGAAG3′; CHOP: Forward: 5′TTGCCCTCTTATTGGTCCAGC3′; Reverse: 5′TAGCGACTGTTCTGTTCCCAC3′.

Quantitative PCR (qRT-PCR) was carried out on a QuantStudio 7 Fast Real-Time PCR System using SYBR Green PCR master mix (Applied Biosystems). The comparative C_t_ method was used to determine relative gene expression using GAPDH to standardize expression levels across all samples. Raw Ct values are displayed in table format in Supplemental Materials (Additional file 5: Supplemental Tables).

### Statistical analysis

Statistical significance for all measurements was determined by both Student’s t test and one-way ANOVA with Newman-Keuls post-test, and *p* < 0.05 was considered statistically significant, with *p < 0.05, **p < 0.01, ***p < 0.001, ****p < 0.0001. Data are represented as mean ± SEM. All statistical analyses were performed using GraphPad Prism software version 5.0.

## Results

### ERAP1 dysfunction enhances NLR-dependent inflammasome responses in macrophages

**Fig. 1:**
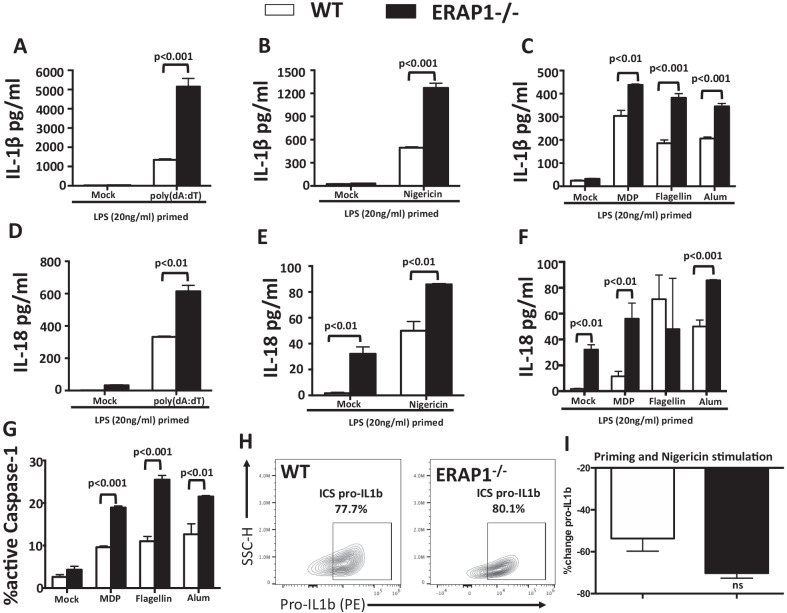
ERAP1 deficient macrophages have increased IL-1β, IL-18, and capsase-1 activation upon inflammasomes activation. Bone marrow-derived macrophages (5 × 10^5^ cells) were plated into 24-well plates. Cells were primed with LPS (20 ng/mL) for 16 h, and then stimulated for another 24 h with alum (1 mg/mL), nigericin (10 ug/mL), MDP (10 ug/mL), flagellin (250 ng/mL), and poly(dA:dT) (8 μM). Production of IL-1β (**A**–**C**) and IL-18 (**D**–**F**) in cell supernatant was performed. Caspase-1 activity (**G**) was evaluated using FLICA flow-based assay. Intracellular pro-IL1β was detected by flow cytometry using Cytek Aurora with various conditions. Baseline mock levels (**H**) as well as percent reduction of pro-IL-1β with priming and nigericin stimulation was compared in WT and ERAP1^−/−^ (**I**). Cells were plated in quadruplicate. Data are expressed as means ± SEM. These figures are representative of four independent experiments

We wished to determine the mechanism linking ERAP1 to regulation of inflammasome pathways; including IL-1β processing and release, using a BMDM-based model. We previously demonstrated that ERAP1 is involved in caspase-1-dependent IL-1β production from human immune cells [6], however the molecular mechanism(s) and the signaling pathway(s) underlying these ERAP1-dependent responses are not understood. To examine if ERAP1 is involved in regulating inflammasome responses, BMDMs derived from WT and ERAP1^−/−^ mice were LPS-primed and stimulated with multiple inflammasome agonists, and subsequent production of IL-1β, IL-18, and caspase-1 activation was evaluated. Consistent with previous reports [21], stimulating WT mice-derived BMDMs with AIM-2 inflammasome agonists (double stranded DNA (dsDNA) and poly(dA:dT)), significantly enhanced IL-1β production (Fig. 1A). Interestingly, we found that stimulation of AIM2 in ERAP1^−/−^ BMDMs produced significantly (*p* < 0.001) higher levels of IL-1β, as compared to WT BMDMs (Fig. 1A). To determine if ERAP1 regulates immune responses derived from activation of other inflammasomes, LPS-primed WT and ERAP1^−/−^ BMDMs were stimulated with NLRP3 (nigericin and alum), NLRP1 (MDP), and NLRC4 (flagellin) agonists. We again detected significantly (*p* < 0.001) higher levels of IL-1β production from ERAP1^−/−^ derived macrophages following nigericin, MPD, flagellin, and alum stimulation, as compared to WT-derived macrophages that were similarly treated (Fig. 1B, C). These results suggest that loss of ERAP1 functions results in exaggerated AIM2, NLRP1, NLRP3, and NLRC4 inflammasome responses. Similar to the IL-1β results, ERAP1^−/−^ macrophages produced higher levels of IL-18 following poly(dA:dT), nigericin, and alum stimulation (Fig. 1D–F). We then investigated if loss of ERAP1 also results in increased caspase-1 activation. Indeed, ERAP1^−/−^ macrophages possessed significantly increased levels of caspase-1 activation after MDP, flagellin and alum stimulations, as compared to identically treated WT macrophages (Fig. 1G). To verify whether increased inflammasome activation was due to mature IL-1β production and not general elevation of baseline pro-IL-1β in ERAP^−/−^, we evaluated basal levels of pro-IL-1β using flow cytometry. We determined that basal levels were not significantly different between WT and ERAP1^−/−^ cells, supporting the notion that ERAP1^−/−^ BMDMs have increased inflammasome activation responses to agonists and therefore greater cleavage of pro-IL-1β into its active, secreted form (Fig. 1H). Although not significant, pro-IL-1β was reduced to a greater extent in ERAP^−/−^ cells, further supporting our data of increased IL-1β processing and release in ERAP-deficient macrophages (Fig. 1I). Together with enhanced inflammasome responses, ERAP1^−/−^ BMDMs also had increased surface expression of macrophage activation markers CD80 and CD86 following various exposures to inflammasome agonists (Additional file 1: Fig. S1).

### Exaggerated cytokine and chemokine responses occur following inflammasome stimulation of ERAP1^−/−^ macrophages

**Fig. 2:**
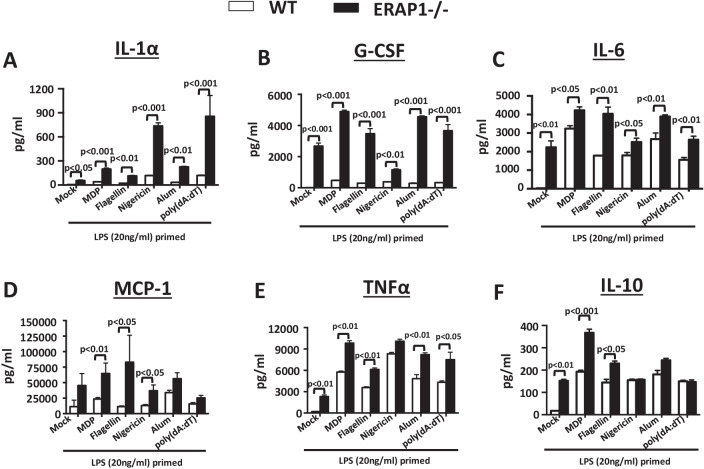
ERAP1 deficiency enhances cytokine and chemokine responses in macrophages following inflammasome stimulation. Bone marrow-derived macrophages (5 × 10^5^ cells) were plated into 24-well plates. Cells were primed with LPS (20 ng/mL) for 16 h, and then stimulated for another 24 h with various NLR inflammasome agonists, as indicated. Cell supernatants were collected, and a 23-plex multiplex assay was performed. Production of IL-1α (**A**), G-CSF (**B**), IL-6 (**C**), MCP-1 (**D**), TNFα (**E**), and IL-10 (**F**) following NLRs stimulation is shown. Cells were plated in quadruplicate. Data are expressed as means ± SEM. These figures are representative of four independent experiments

We next determined the impact that ERAP1 deficiency has on inflammasome-regulated cytokine and chemokine responses. Cytokines present in supernatants derived from inflammasome-activated WT and ERAP1^−/−^ BMDM cultures were quantified using a 23-plex multiplex assay, as previously described [6]. We observed significant increases in the production of IL-1α (Fig. 2A), G-CSF (Fig. 2B), IL-6 (Fig. 2C), MCP-1 (Fig. 2D), and TNFα (Fig. 2E) from ERAP1^−/−^ macrophages, as compared to WT macrophages identically treated with MDP, flagellin, nigericin, alum, and poly(dA:dT). IL-10 levels were also significantly increased in MDP and flagellin (but not nigericin, alum, and poly(dA:dT)) stimulated ERAP1^−/−^ BMDMs, as compared to WT BMDMs (Fig. 2F). It is important to note that ERAP1^−/−^ macrophages were also more sensitive to LPS-priming as compared to WT macrophages, suggesting an inherently increased sensitivity of ERAP1^−/−^ BMDMs to TLR4 stimulation (Fig. 2A–F). Following nigericin stimulation, a potent inducer of the NLRP3 inflammasome, multiplexed assays confirmed increased production of several cytokines including IL-1α, MCP-1, TNFα, MIP-1, IL-2, IL-13, and IL-9 from ERAP1^−/−^ derived macrophages, as compared to WT-derived macrophages (Additional file 2: Fig. S2). Together these data suggest that ERAP1 deficient macrophages are inherently more susceptible to innate immune stimulation and have exaggerated proinflammatory cytokine responses after exposure to inflammasome agonists.

### The effect of ERAP1 deficiency on TLR signaling pathways

**Fig. 3:**
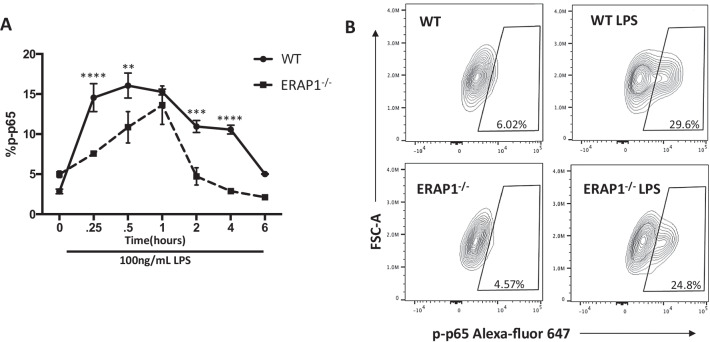
ERAP1 deficiency causes enhanced inflammatory responses through an NF-κB independent mechanism. Bone marrow derived macrophages (5 × 10^5^ cells) were plated into 24 well plates, and activated with 100 ng/mL LPS for various timepoints (15 min, 30 min, 1 h, 2 h, 4 h, 6 h). Cells were immediately fixed, permeabilized and stained for p-p65 using BD Phosphoflow Perm Buffer per manufacturer’s guidelines. p-P65 activation was assessed with Cytek Aurora followed by analysis in FlowJo (**A**). Representative images of flow cytometry for mock and 1 h LPS stimulation (**B**) Data are representatives of two independent experiments. Data are expressed as means ± SEM

As ERAP1 deficient cells are highly sensitive to LPS stimulation alone, we tested whether ERAP1^−/−^ deficiency modulates TLR signaling, specifically via the NF-κb pathway [22]. NF-κB is a known activator of proinflammatory cytokines such as IL-2, IL-6, IL-12, and TNFα and enhanced NF-κB activity could explain the increased proinflammatory cytokine secretion, albeit non-specifically. [23]. To assess whether ERAP1^−/−^ BMDMs have enhanced NF-κB activation, we evaluated p65 phosphorylation, the active subunit of NF-κB, by a phospho-flow-based assay [24]. To our surprise, we found that LPS treated ERAP1^−/−^ BMDMs had less phosphorylated p65 than identically treated WT BMDMs (Fig. 3A, B) at various timepoints. This effect on TLR signaling is independent of cell viability, as both ERAP1-deficient and WT cells are equally susceptible to cell death by stimuli (data not shown). This data suggests that ERAP1 may be regulating proinflammatory responses through an NF-κB independent mechanism.

### MHC-I dysregulation in ERAP1 deficient immune cells

**Fig. 4:**
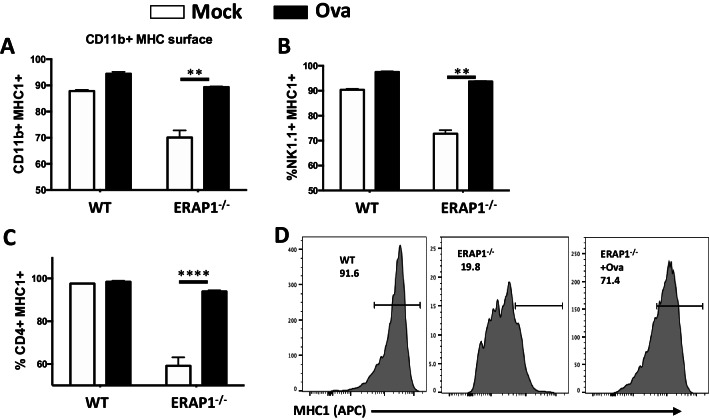
MHC-I surface expression is reduced on ERAP1^−/−^ BMDMs. Total splenocytes were isolated from WT and ERAP1^−/−^ and incubated with or without 25ug Synficol (Ova). Cells were incubated for 18 h. Cell surface MHC-I (APC) expression was analyzed in CD4 + , NK1.1 + , and CD11b + cells by flow cytometry (**A**–**C**). BMDMs were also cultured in the same manner and MHC-I surface expression was assessed (**D**). This figure is a representative of 3 independent experiment. Data are expressed as means ± SEM. *p < 0.05, **p < 0.01, ****p < 0.00001 significantly different from mock

Lack of ERAP1 functions can lead to deficient peptide loading onto awaiting MHC-I molecules, resulting in unfolded protein responses that may lead to ER stress [17, 19, 25–27]. Prior studies utilizing ERAP1^−/−^ mice transgenically expressing human ERAP1 variants, as well as other studies, have supported that alterations in the enzymatic activity of ERAP1 diminishes the production of appropriately sized peptides for MHC-I loading, potentially resulting in the ER accumulation of empty MHC-I heavy chains [5, 6]. Furthermore, HLA-B27 (the HLA allele most associated with AS, and found to be in epistasis in AS patients) misfolding has been linked to activation of the unfolded protein response (UPR) and production of proinflammatory cytokines via NF-κB, JNK1/2, p38 and/or Erk1/2 activation [17, 25]. This may be due to multiple reasons such as lack of appropriate ERAP1 aminopeptidase activity reducing the pool of appropriately sized peptides available for MHC-I loading, leading to aggregation of incorrectly folded MHC-I molecules within the cell and reduced MHC-I expression on the cell surface [7, 28]. Indeed, we show here that MHC-I surface expression was reduced on various ERAP1^−/−^ immune cells, as compared to similarly assessed WT cells (Fig. 4). However, this reduction could be corrected with the provision of supranormal amounts of ovalbumin (ova) derived SINFEKL peptide, a procedure that bypasses the need for ERAP1 dependent processing, and thereby restores surface levels of MHC-I in ERAP1 deficient cells [27, 29].

### Increased expression of ER stress genes in ERAP1 deficient macrophages

**Fig. 5:**
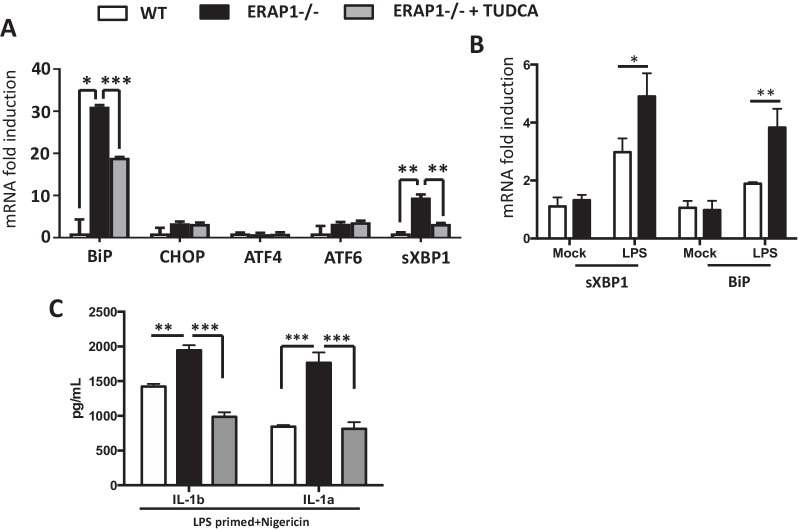
ERAP1 is a regulator of ER stress. Bone marrow-derived macrophages (2 × 10^6^ cells) were plated into 6-well plates. Cells were pretreated with 500 ug/mL of TUDCA for 48 h and were then stimulated with LPS (25 ng/mL) for 18 h (**A**). Cells were harvested for RNA isolation. mRNA levels of ATF6, ATF4, BiP, CHOP, sXBP1 and GAPDH were evaluated. BMDMs were primed with LPS overnight, incubated with TUDCA the next day, and then stimulated with nigericin for an additional 16 h. Supernatant was collected and 23-bead Bioplex was run for cytokine examination of IL-1β and IL-1α (**C**). CD4^+^ T cells were isolated from total splenocytes, pooled, and plated at 3 × 10^6^ cells per well into 6-well plates. Cells were stimulated with LPS (20 ng/mL) for approximately 12 h. Cells were harvested for RNA isolation. Cells were collected from 5 WT and 4 ERAP1^−/−^ cells and kept separate during the experiment. mRNA levels of ER stress genes BiP and sXBP1 (**B**) was assessed. Raw Ct values from representative experiments are presented in Supplemental Information (Additional file 5: Supplemental Tables). Data are expressed as means ± SEM. These figures are representative of four independent experiments

It is also well known that a possible downstream effect of protein accumulation in the ER is ER stress-dependent NLRP3 inflammasome activation, as the NLRP3 inflammasome is indeed a sensor of ER stress [17, 18, 26]. Given this, together with decreased surface MHC-I expression in ERAP^−/−^ cells, we hypothesized that increased innate signaling in ERAP deficient cells may be due to the presence of ER stress. More specifically, that the increased sensitivity of ERAP1^−/−^ macrophages to inflammasome agonists might be associated with the presence of increased ER stress precipitated by lack of ERAP1-dependent peptide trimming functions. Using LPS-primed WT and ERAP1^−/−^ BMDMs in the presence or absence of the ER stress inhibitor tauroursodeoxycholic acid (TUDCA), we quantified ER stress-associated gene expression by qRT-PCR. Interestingly, ERAP1^−/−^ BMDMs had significant increases (30-fold) in ER stress chaperone BiP mRNA levels, as compared to BiP levels in WT BMDMs cultured under these same conditions (Fig. 5A). Also, we detected increased expression of the spliced variant of the X-box-binding protein 1(sXBP1) in ERAP1^−/−^ BMDMs, as compared to WT BMDMs (Fig. 5A). Importantly, TUDCA significantly decreased these responses (Fig. 5A). Protein ER stress levels of the activated form of IRE-1α, p-IRE1α, were also increased in ERAP1-deficient BMDMS in comparison to WT BMDMs, and remained at higher expression levels over time (Additional file 3: Fig. S3).

As mentioned previously, a possible downstream effect of ER stress is NLRP3 inflammasome activation [26]. The NLRP3 inflammasome is a sensor of ER stress, which can utilize a non-canonical inflammasome pathway independent of ASC speck formation [26]. We found no significant changes in ASC speck formation between WT and ERAP1^−/−^ BMDMs, revealing that ER stress dependent enhanced inflammasome responses due to ERAP1 deficiency may also be occurring in an ASC speck independent manner (Additional file 4: Fig. S4).

We further studied associations between ERAP1-dependent inflammatory responses and ER stress in additional immune cell types, particularly in subsets found to be altered in ERAP1^−/−^ mice such as CD4^+^ T cells [8]. We confirmed that significantly elevated levels of both BiP and sXBP1 were present in LPS-stimulated ERAP1^−/−^ CD4^+^ T cells in comparison to similarly treated WT CD4^+^ T cells (Fig. 5B).

We next wished to study whether this induction in ER stress genes was linked with inflammasome activity. Importantly, when ER stress was inhibited with TUDCA, this also reverted the enhanced IL-1β and IL-1α cytokine production in ERAP1^−/−^ cells to WT levels (Fig. 5C), further supporting the importance of ER stress in ERAP1-modulated proinflammatory responses. These data suggest that ERAP1’s role in inflammasome regulation is associated with increased ER stress (Additional file 5: Table S1).

### Increased proinflammatory cytokine and chemokine responses in colon tissues of ERAP1 deficient mice following DSS administration

**Fig. 6:**
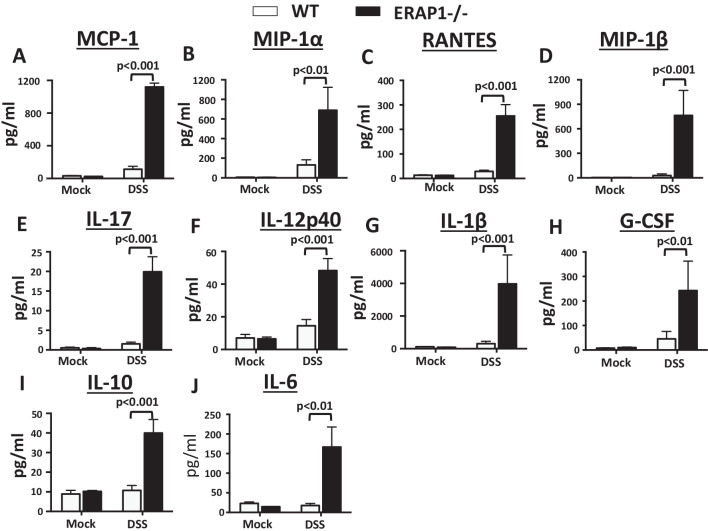
ERAP1 modulates colonic inflammatory cytokines and chemokines during DSS-induced colitis. Male WT and ERAP1^−/−^ mice (n = 7) were treated with a 3% DSS solution in drinking water for 7 days. Colon tissues were collected at day 7 following DSS treatment. Colon homogenates were prepared as described in “Materials and methods” section. **A**–**J** A 23-plex multiplex analysis on colon lysate supernatants was used to evaluate the concentration of the indicated cytokines and chemokines. Data are expressed as means ± SEM

Previously, we demonstrated that ERAP1^−/−^ mice have enhanced susceptibility to DSS-induced colitis [8]. DSS is not only a well-studied induction model for colitis, but DSS can also induce caspase-1 cleavage via activation of the NLRP3 inflammasome [30]. As expected, colon homogenates from DSS-treated ERAP1^−/−^ mice contain significantly higher levels of several proinflammatory cytokines and chemokines, as compared to identically treated WT mice (Fig. 6). Specifically, we observed dramatic increases in the production levels of several CC chemokines (MCP-1 (*p* < 0.001), MIP-1α (*p* < 0.01), RANTES (*p* < 0.001), and MIP-1β) (Fig. 6A–D), Th-17- (IL-17α (*p* < 0.001)) (Fig. 6E), Th-1- (IL-12p40 (*p* < 0.001), IL-1β, and G-CSF) (Fig. 6F–H), and Th-2-skewing cytokines (IL-10 (*p* < 0.001) and IL-6) (Fig. 6I, J) in colon homogenates of DSS treated ERAP1^−/−^ mice, as compared to WT controls. We also noted increased trends towards increased production of other proinflammatory cytokines including IL-1α, IL-2, GM-CSF, and EOTAXIN in colon homogenates of DSS-treated ERAP1^−/−^ mice as compared to DSS-treated WT controls; however, these trends did not reach statistical significance (data not shown). Together, these results suggest that ERAP1 deficiency also results in exaggerated colonic cytokine and chemokine responses during inflammasome (DSS)-induced intestinal inflammation.

### Enhanced pro-inflammatory cytokines with Monosodium Urate Crystal

**Fig. 7:**
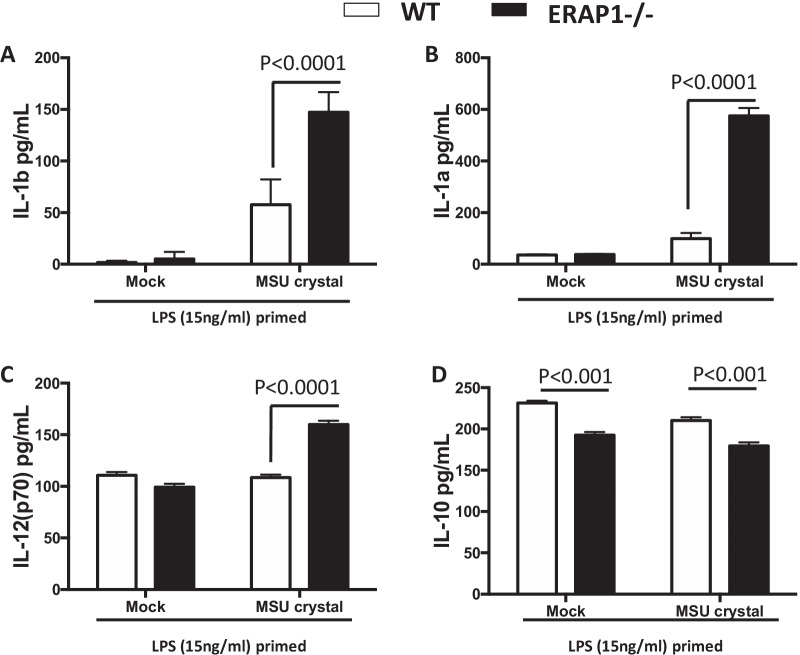
ERAP1 deficient macrophages are sensitive to NLRP3 activation by MSU crystals. Bone marrow-derived macrophages (2 × 10^6^ cells) were plated into 6-well plates. Cells were primed overnight with 15 ng/mL LPS, and then stimulated with or without 100 ug/mL MSU crystals for approximately 18 h. Cell supernatant was collected, and cytokine quantification was done by 23-plex Bioplex assay (**A**–**D**). Data is representative of two independent experiments and expressed as means ± SEM

As ERAP1 polymorphisms have been linked with inflammatory arthritic disease ankylosing spondylitis [1, 31, 32], we wished to study the role of ERAP1 in relation to the NLRP3 inflammasome specifically in arthritis-linked disorders. Monosodium urate (MSU) crystals are both potent NLRP3 inflammasome inducers as well as mediators of other inflammatory diseases such as gout [33]. In addition to gout, MSU crystal deposits were discovered in the spines of patients with ankylosing spondylitis without gout, and interestingly, these MSU crystals were correlated with more severe sacroiliac joint disease [34]. Given that we previously published ERAP1 deficient mice have enhanced bony fusions in a phenotype similar to ankylosing spondylitis, and our results demonstrating that ERAP1-deficient BMDMs have elevated IL-1β production, we wished to study the impact of MSU crystals on ERAP^−/−^ cells. We determined that ERAP1^−/−^ BMDMs not only had increased production of IL-1β (Fig. 7A), but also increased IL-1α and IL-12 secretion (Fig. 7B, C), and conversely less IL-10 production (Fig. 7D) after exposure to MSU crystals. Although the clinical connection between MSU crystals, the inflammasome, and ankylosing spondylitis are not well established, this data further supports ERAP1’s important role in general inflammasome and innate immune regulation.

## Discussion

Innate immune activation is a common denominator in the pathophysiology of autoimmune diseases, and is many times characterized by increases in immune mediators, such as cytokines like TNFα and IL6, and exaggerated inflammasome activity [1, 5, 6, 16]. ERAP1 gene polymorphisms causing improper ERAP1 functions have been linked to several autoimmune diseases [1, 35–37]; however, the exact molecular mechanisms underlying these associations are yet to be defined. Our previous studies illustrated that ERAP1 polymorphisms in human cells enhance innate signaling, and recognize this as a possible cause for enhanced susceptibility to ERAP1 linked autoimmune diseases [6]. By utilizing in vitro and in vivo murine models, we can further study the role of ERAP1 in autoimmune diseases. Our results here confirm that loss of ERAP1 functions resulted in increased sensitivity to inflammasome stimulation, a phenomenon that results in exaggerated caspase-1 activation and production of proinflammatory cytokines and chemokines following AIM-2, NLRP1, NLRP3, and NLRC4 inflammasome activation. Since flagellin might also activate TLR5 pathway, our results suggest that ERAP1 deficiency might also impact TLR5 signaling pathway [38]. Interestingly, we discovered that the mechanism by which ERAP1 impacts the NLRP3 inflammasome is by an ASC speck-independent manner, a finding supported by recent studies describing NLRP3 and ASC speck functions as distinct processes [39].

The NF-κB pathway is one of the most well studied signaling pathways in immunology and is an important driver of innate immune responses. Activation of NF-κB can induce multiple cytokines, chemokines, and genes all involved in a proinflammatory response [40, 41]. TLR4 activation by LPS can induce NF-κB activity through the adaptor molecule MYD88 [42, 43], and since we found enhanced cytokine production in ERAP1-deficient BMDMs with LPS priming alone, NF-κB activity was important for us to assess. In our studies we determined that NF-κB activation was actually statistically significantly reduced in ERAP1^−/−^ BMDMs as compared to WT. This data suggests that although ERAP1^−/−^ cells have enhanced proinflammatory cytokine and chemokine secretion, this may be occurring through an NF-κB independent mechanism. As heightened induction of NF-κB in ERAP1^−/−^ cells does not seem to be occurring, the potential importance of the inflammasome in these cells became paramount to determine.

One possibility as to why ERAP1 functions may be necessary to modulate the multiprotein inflammasome complexes may be that ERAP1 regulates inflammasome responses by directly interacting with the inflammasome complex in the ER, or other compartment of the cell. Intriguingly, resting NLRs, such as NLRP3, are also known to localize to ER structures, providing a direct mechanism as to how these pathways are modulated by lack of appropriate ER peptidase functions caused by mutations in ER proteins such as ERAP1 [44].

ER stress responses activate proinflammatory pathways [44–46] and previous studies have linked ER stress as a cause for DSS-induced colitis in vivo specifically, and in the pathophysiology of IBD in general [45, 46], both of which also share a strong correlation with NLRP3 inflammasome activation [30]. Our current results confirm that ERAP1 is important for maintaining proper homeostasis of the ER, as evidenced by the increased expression levels of important ER stress-associated genes in the absence of a functional ERAP1 protein. Not only were ER stress genes elevated in ERAP1^−/−^ BMDMs, but also in ERAP1^−/−^ CD4^+^ T cells as compared to WT cells. Interestingly, prevention of ER stress prevented exaggerated inflammasome activation in stimulated ERAP1^−/−^ cells, linking loss of ERAP1 to both ER stress induction and activation of the NLRP3 inflammasome.

Together, our results suggest that ERAP1^−/−^ BMDMs may be inherently predisposed to exaggerated inflammasome responses upon encounter with typical inflammasome agonists as a result of increased ER stress being constitutively present in these cells. As one reason amongst several, this may be due to lack of efficient processing of MHC-I destined peptides and translocation, as evidenced by loss of surface MHC-I on ERAP1 deficient cells [4, 5, 7]. Here we show that MHC-I surface expression is indeed reduced on multiple immune ERAP1^−/−^ cell types, and that this reduction can be overcome when incubated with an ovalbumin derived peptide, which is not reliant on ERAP1 processing for loading onto MHC-I. These data suggest that enhanced ER stress may be present in multiple cell types when ERAP1 functions in the ER are inadequate.

Patients with autoimmune disease such as AS have a high likelihood to also suffer from inflammatory bowel diseases (IBD) such as UC. We demonstrated here that loss of ERAP1 also results in excessive proinflammatory cytokine and chemokine responses during DSS-induced colitis. For example, the NLR inflammasome-mediated production of IL-1β and IL-18 is known to increase in the inflamed mucosa of UC patients [47], and our data suggest that loss of ERAP1 results in excessive production of IL-1β and IL-18 from activated inflammasomes ex vivo. This was independent of baseline pro-IL-1β levels. Importantly, NLRP3 polymorphisms that impact IL-1β and IL-18 production have also been previously associated with an increased susceptibility to UC in humans [48]. In addition, increased caspase-1 activation and IL-1β production by the constitutively active autoimmune disease-associated variants of NLRP3 were also linked to autoimmune diseases [49], indicating a crucial need for appropriate NLRP3 activation and IL-1β production. Together, our current data reveal that the presence of ERAP1 is important for maintaining balanced IL-1β levels during episodes of intestinal inflammation.

There is clear overlap between ERAP1-regulated cytokine and chemokine responses and the cytokine and chemokine profile that is commonly present in UC patients. For example, an increased production of IL-6 and its soluble receptor (sIL-6R) is commonly observed in UC patients with active inflammation [50]. Notably, in our studies we found that ERAP1 plays an important role in regulating IL-6 in the colons of DSS-treated ERAP1^−/−^ mice. ERAP1 is also known to cause shedding of IL-6 [51], further linking ERAP1 and regulation of IL-6 in colitis. Importantly, this data overall shows ERAP1’s critical role in innate immune signaling. Although IL-1β is increased in ERAP1^−/−^ colons, we found an overwhelming proinflammatory cytokine profile, indicating that ERAP1 may regulate global inflammatory responses not solely specific to the inflammasome.

In the past few years, studies have found that patients with AS also have MSU crystal deposition in their sacroiliac joints [34]. As MSU crystals are a known inducer of the NLRP3 inflammasome, we wished to further study their role in relation to ERAP1 ex vivo. As expected, ERAP1-deficient macrophages showed enhanced inflammasome activity in response to NLRP3 activation by MSU crystals. Other pro-inflammatory cytokines were elevated with the stimuli in ERAP1^−/−^ BMDMs including IL-12, but also the anti-inflammatory cytokine IL-10 was reduced, further supporting a pro-inflammatory signature in ERAP1^−/−^ BMDMs. Together with the DSS studies shown here, this data further underpins the importance of ERAP1 in inflammatory diseases and begins to investigate how enhanced inflammation in ERAP1-deficient animals may be occurring, potentially through exaggerated activation of the inflammasome.

In summary, we demonstrated that ERAP1 is a regulator of innate inflammatory cytokines and the inflammasome. We discovered here that the presence of increased ER stress responses in ERAP1 deficient cells may explain why lack of ERAP1 function results in excessive activation of several caspase-1-activating inflammasomes and exaggerated production of cytokines following innate immune system activation. Our data suggest that the strong genetic associations between ERAP1 polymorphisms and various autoimmune diseases may be linked to ERAP1’s role in innate immune regulation, including regulating proinflammatory cytokine and chemokine responses during inflammasome activation. Here we also suggest the important connection between ERAP1 and ER stress, uncovering new potential targets for the treatment, and/or prevention of ERAP1 linked autoimmune diseases. Future studies into the definitive mechanism for which signaling molecules or chaperones are triggering inflammasome activation in ERAP1^−/−^ cells should be explored.

## Supplementary Information

**Additional file 1.** **Supplemental Figure 1:** Characterization of macrophages with multiple inflammasome agonists.Bone marrow-derived macrophages (5 × 105 cells) were plated into 24-well plates, and then cells were primed with LPS (20 ng/ml) for 16 hours. Cells were then stimulated foranother 24 hours with various NLR inflammasome agonists, as indicated. Cells were stained with Pacific Blue conjugated-CD80 (C, D) and APC conjugated anti-CD86 (E,F) and flow cytometry was completed. Data are expressed as means ± SEM. p < 0.01, p < 0.001, significantly different from mock.

**Additional file 2.** **Supplemental Figure 2:** Increased cytokine and chemokine production from ERAP1-/- macrophages following NLRP3 inflammasome activation. Heat map depicting the top 12 upregulated and downregulated cytokines by ERAP1. Bone marrow-derived macrophages (BMDMs) from C57 WT and ERAP1-/- mice were primed with LPS (20 ng/mL) for 12 hours. Cells were then stimulated with nigericin (10 ug/mL) for an additional 20 hours. Supernatants were collected and analyzed used in a 23-plex multiplex assay using Bioplex.

**Additional file 3.**  **Supplemental Figure 3:** p-IRE1a expression with NLRP3 inflammasome stimulation. Bone marrow-derived macrophages (2 × 106 cells) were plated into 6-well plates, and then cells were primed with LPS (15 ng/ml) for 12 hours. Cells were then stimulated for various timepoints with Nigericin (10 uM). BMDMs were collected, protein was isolated and western blot was run for p-IRE1a and loading control.

**Additional file 4.** **Supplemental Figure 4:** ASC speck formation analysis with NLRP3 inflammasome stimulation. Bone marrow-derived macrophages (1 × 106 cells) were plated into 6-well plates, and then cells were primed with LPS (15 ng/ml) for 12 hours. Cells were then stimulated for 30 minutes with Nigericin (Nig.). Cells were stained with Alexa 488-conjugated ASC and DAPI nuclear stain, followed by sample collection and analysis via ImageStream X.

**Additional file 5.** **Supplemental Tables:** Raw Ct values collected from representative experiments presented in Figure 5.

## Data Availability

The datasets analyzed during the current study are available from the corresponding author on reasonable request.
