# Supplementary material for: ERAP1 is a critical regulator of inflammasome-mediated proinflammatory and ER stress responses
Source: BMC Immunol. 2022 Mar 4;23:9. doi: 10.1186/s12865-022-00481-9 (PMC8895631; doi:10.1186/s12865-022-00481-9)
Supplement: Supplementary file 1 — Additional file 1. Supplemental Figure 1: Characterization of macrophages with multiple inflammasome agonists.Bone marrow-derived macrophages (5 × 10^5^ cells) were plated into 24-well plates, and then cells were primed with LPS (20 ng/ml) for 16 hours. Cells were then stimulated foranother 24 hours with various NLR inflammasome agonists, as indicated. Cells were stained with Pacific Blue conjugated-CD80 (C, D) and APC conjugated anti-CD86 (E,F) and flow cytometry was completed. Data are expressed as means ± SEM. p < 0.01, p < 0.001, significantly different from mock. [file 12865_2022_481_MOESM1_ESM.pptx]

## Slide 1
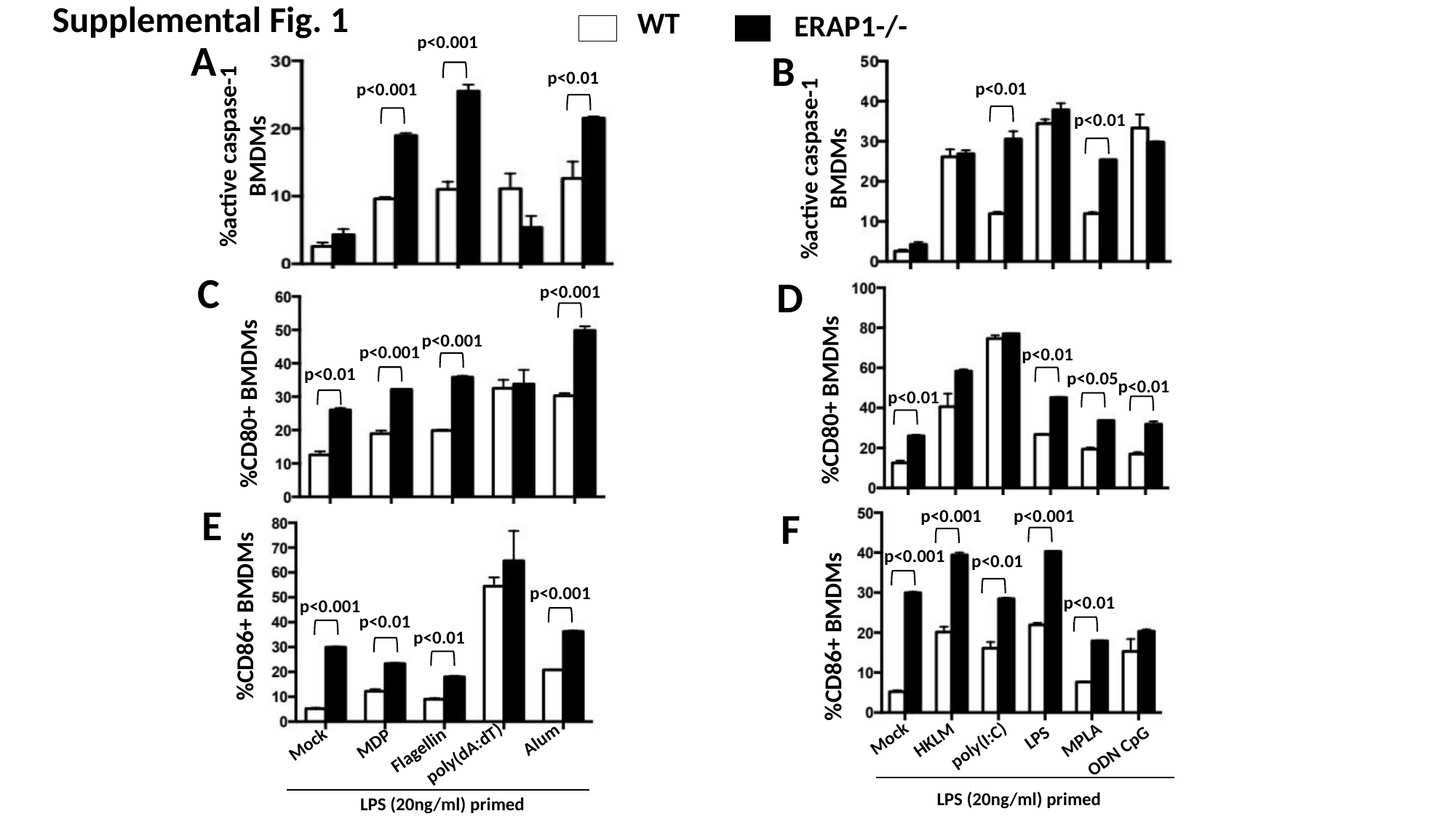

Supplemental Fig. 1
WT
ERAP1-/-
p<0.001
A
B
p<0.01
p<0.01
p<0.001
p<0.01
%active caspase-1
 BMDMs
%active caspase-1
 BMDMs
C
D
p<0.001
p<0.001
p<0.001
p<0.01
p<0.01
p<0.05
p<0.01
%CD80+ BMDMs
p<0.01
%CD80+ BMDMs
E
F
p<0.001
p<0.001
p<0.001
p<0.01
p<0.001
p<0.01
p<0.001
%CD86+ BMDMs
p<0.01
%CD86+ BMDMs
p<0.01
LPS
Mock
Alum
MPLA
HKLM
MDP
Mock
poly(I:C)
Flagellin
ODN CpG
poly(dA:dT)
LPS (20ng/ml) primed
LPS (20ng/ml) primed
