# Supplementary figures and images for: ERAP1 is a critical regulator of inflammasome-mediated proinflammatory and ER stress responses
Source: BMC Immunol. 2022 Mar 4;23:9. doi: 10.1186/s12865-022-00481-9 (PMC8895631; doi:10.1186/s12865-022-00481-9)

## Slide 1
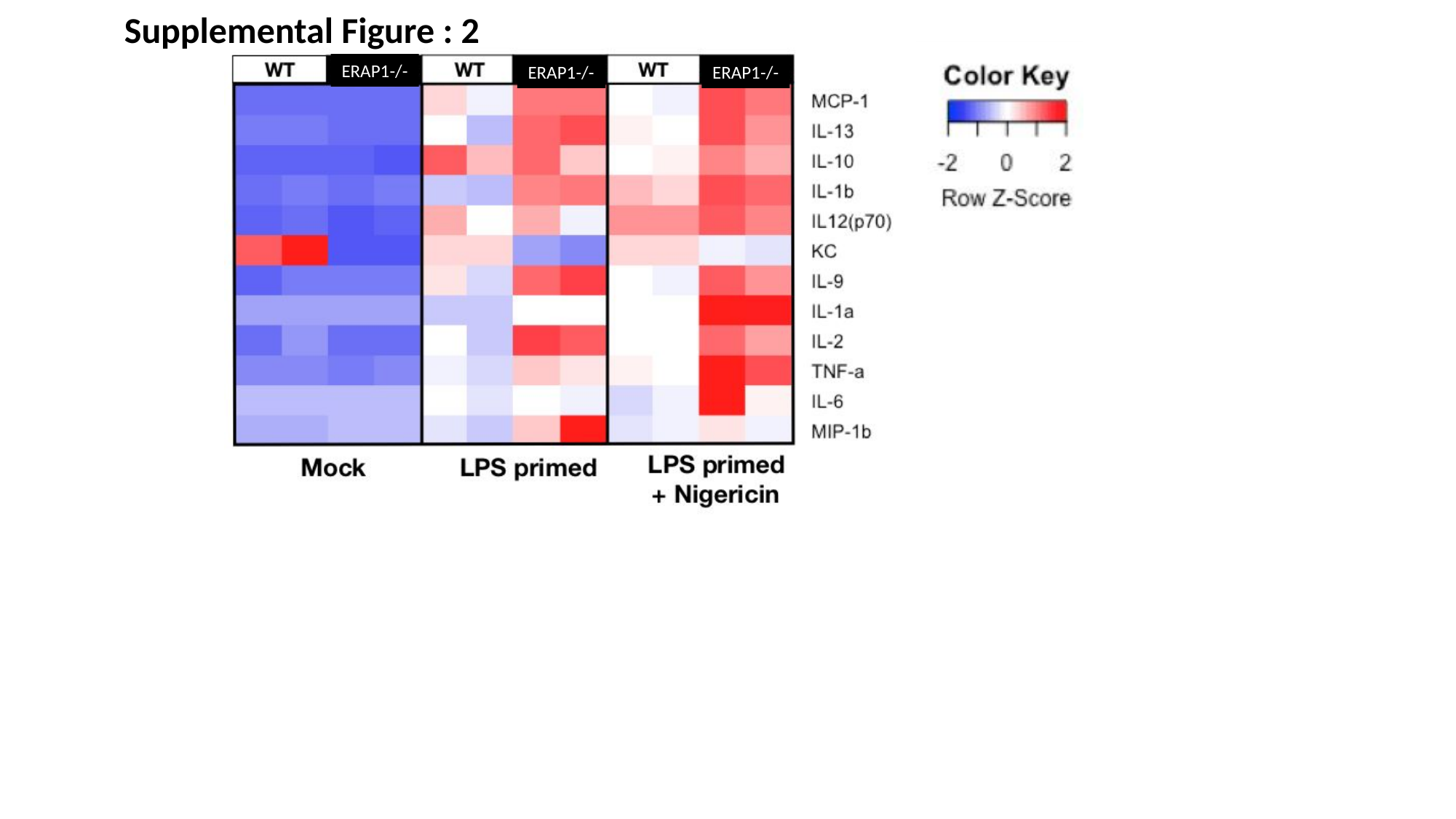

Supplemental Figure : 2
ERAP1-/-
ERAP1-/-
ERAP1-/-

Supplement: Supplementary file 2 — Additional file 2. Supplemental Figure 2: Increased cytokine and chemokine production from ERAP1-/- macrophages following NLRP3 inflammasome activation. Heat map depicting the top 12 upregulated and downregulated cytokines by ERAP1. Bone marrow-derived macrophages (BMDMs) from C57 WT and ERAP1-/- mice were primed with LPS (20 ng/mL) for 12 hours. Cells were then stimulated with nigericin (10 ug/mL) for an additional 20 hours. Supernatants were collected and analyzed used in a 23-plex multiplex assay using Bioplex. [file 12865_2022_481_MOESM2_ESM.pptx]

## Slide 1
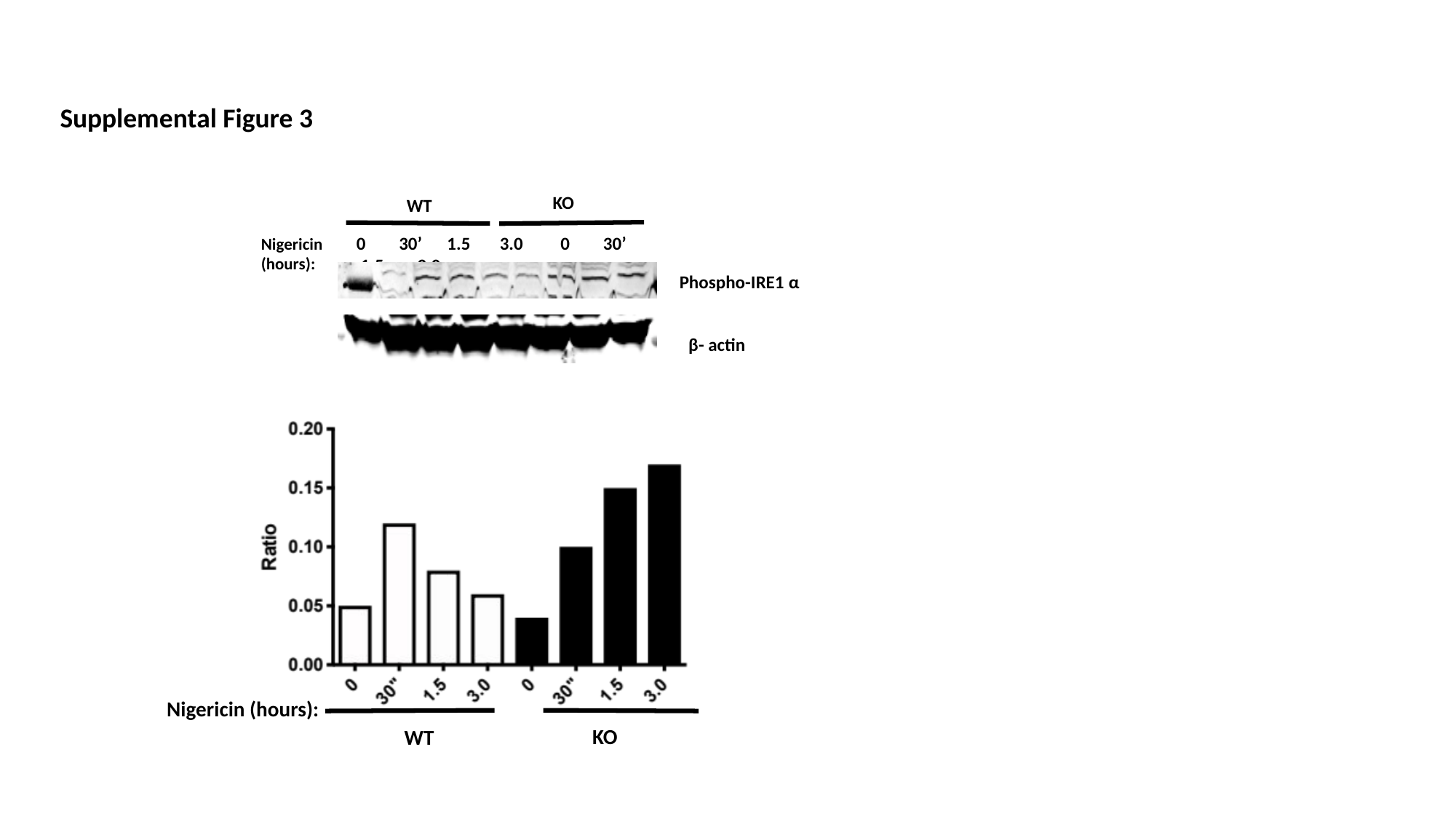

Supplemental Figure 3
KO
WT
0 30’ 1.5 3.0 0 30’ 1.5 3.0
Nigericin (hours):
Phospho-IRE1 α
β- actin
Nigericin (hours):
KO
WT

Supplement: Supplementary file 3 — Additional file 3. Supplemental Figure 3: p-IRE1a expression with NLRP3 inflammasome stimulation. Bone marrow-derived macrophages (2 × 10^6^ cells) were plated into 6-well plates, and then cells were primed with LPS (15 ng/ml) for 12 hours. Cells were then stimulated for various timepoints with Nigericin (10 uM). BMDMs were collected, protein was isolated and western blot was run for p-IRE1a and loading control. [file 12865_2022_481_MOESM3_ESM.pptx]

## Slide 1
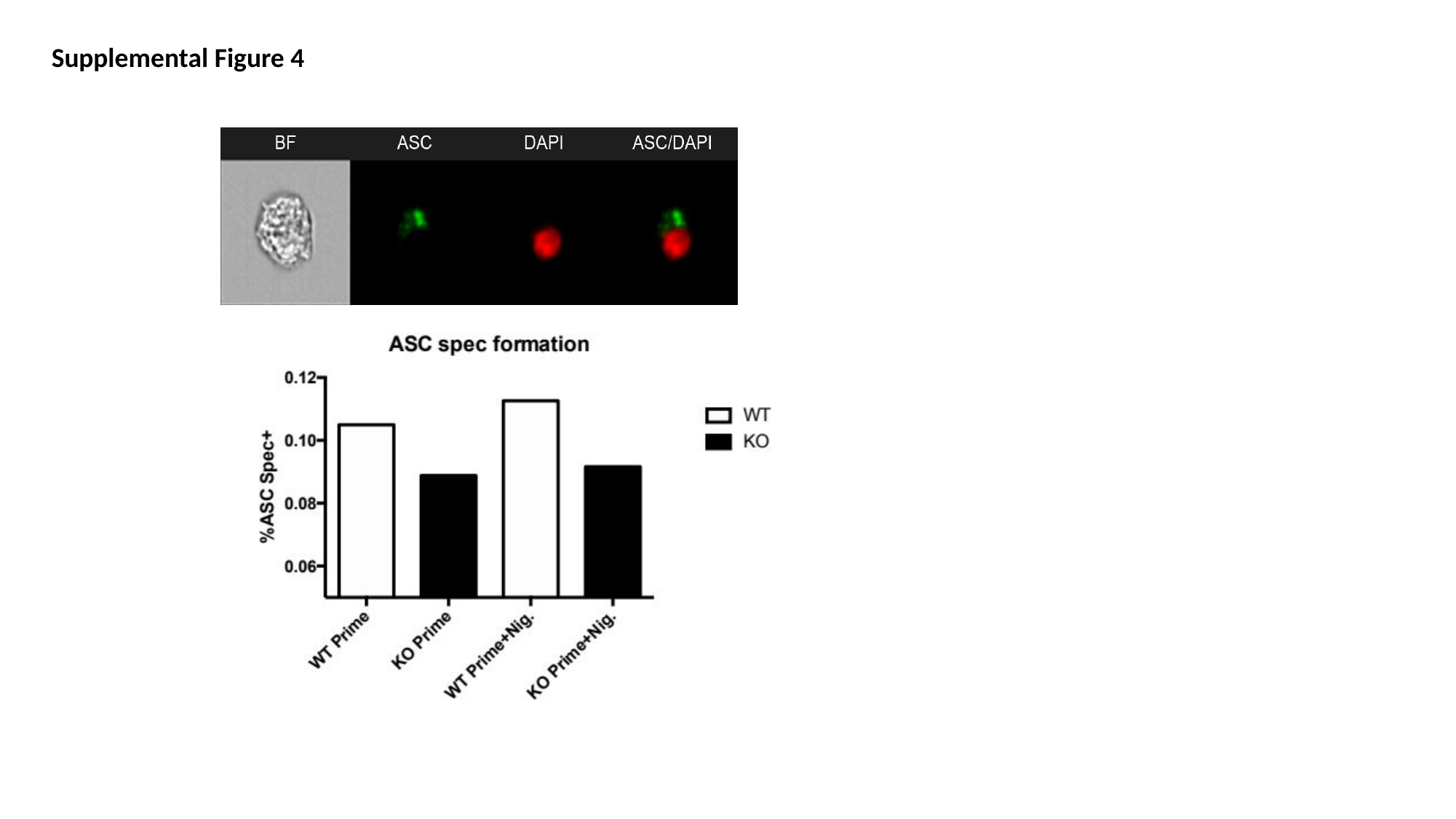

Supplemental Figure 4

Supplement: Supplementary file 4 — Additional file 4. Supplemental Figure 4: ASC speck formation analysis with NLRP3 inflammasome stimulation. Bone marrow-derived macrophages (1 × 10^6^ cells) were plated into 6-well plates, and then cells were primed with LPS (15 ng/ml) for 12 hours. Cells were then stimulated for 30 minutes with Nigericin (Nig.). Cells were stained with Alexa 488-conjugated ASC and DAPI nuclear stain, followed by sample collection and analysis via ImageStream X. [file 12865_2022_481_MOESM4_ESM.pptx]
