# Supplementary material for: ERAP1 is a critical regulator of inflammasome-mediated proinflammatory and ER stress responses
Source: BMC Immunol. 2022 Mar 4;23:9. doi: 10.1186/s12865-022-00481-9 (PMC8895631; doi:10.1186/s12865-022-00481-9)
Supplement: Supplementary file 5 — Additional file 5. Supplemental Tables: Raw Ct values collected from representative experiments presented in Figure 5. [file 12865_2022_481_MOESM5_ESM.pdf]

Supplemental Tables: Raw Ct values collected from representative experiments presented in Figure 5.

Fig. 5A Raw Ct Values

	GAPDH	BiP	CHOP	ATF4	ATF6	sXBP1
WT1 Mock	17.787	29.817	28.963	20.223	26.644	27.798
WT2 Mock	17.646	23.208	26.254	19.789	23.075	27.242
KO1 Mock	14.918	18.746	22.985	17.497	20.263	21.543
KO2 Mock	15.414	19.262	23.586	17.963	20.911	21.897
KO TUDCA 1	14.531	18.879	22.623	16.851	19.795	22.406
KO TUDCA 2	14.088	18.539	22.438	16.548	19.595	22.413
KO TUDCA 3	14.631	18.925	22.765	17.135	19.666	22.748

Fig. 5B Raw Ct Values

	GAPDH CT value	BiP CT value	sXBP1 CT value
WT1 Mock	17.71914	21.14097	25.509
WT2 Mock	18.9938	22.280113	26.478
WT3 Mock	18.404955	22.876427	27.487
KO1 Mock	18.581673	23.237532	26.446
KO2 Mock	19.029003	22.942194	27.064
KO3 Mock	18.337036	22.761652	26.189
KO4 Mock	18.420649	21.209301	25.657
WT1 LPS	18.053438	20.834082	24.648617
WT2 LPS	18.957941	21.706064	25.133324
WT3 LPS	18.41314	21.301208	25.38803
KO1 LPS	19.154375	21.640118	25.173912
KO2 LPS	19.257992	21.16446	24.51236
KO3 LPS	18.672302	20.434326	24.744936
KO4 LPS	18.341055	19.605177	24.483255
